# Aktuelle Glaukomchirurgie

**DOI:** 10.1007/s00347-020-01146-x

**Published:** 2020-07-06

**Authors:** Esther M. Hoffmann, Fritz Hengerer, Karsten Klabe, Marc Schargus, Hagen Thieme, Bogomil Voykov

**Affiliations:** 1 Augenklinik und Poliklinik, Universitätsmedizin Mainz, Mainz, Deutschland; 2 Bürgerhospital Frankfurt a. M., Frankfurt a. M., Deutschland; 3 Breyer – Kaymak – Klabe Augenchirurgie, Düsseldorf, Deutschland; 4 Asklepios Augenklinik Nord-Heidberg Hamburg, Hamburg, Deutschland; 5 Universitäts-Augenklinik Düsseldorf, Düsseldorf, Deutschland; 6 Universitätsaugenklinik Magdeburg, Magdeburg, Deutschland; 7 Universitätsaugenklinik Tübingen, Tübingen, Deutschland

**Keywords:** Glaukom, Trabekulektomie, Minimal-invasive Glaukomchirurgie, Mitomycin C, Zieldruck, Glaucoma, Trabeculectomy, Minimally invasive glaucoma surgery, Mitomycin C, Target pressure

## Abstract

**Hintergrund:**

Bei der Behandlung von Glaukompatienten sind die Hauptziele die Bewahrung der Sehfähigkeit und Aufrechterhaltung einer möglichst hohen Lebensqualität bei volkswirtschaftlich akzeptablen Kosten. Daher ist es wichtig, jeden einzelnen Patienten sorgfältig zu evaluieren, um einen individuellen Behandlungsansatz zu erarbeiten.

**Material/Methoden:**

Basierend auf den aktuellen Erkenntnissen, werden in der Übersicht die Vor- und Nachteile der medikamentösen Glaukomtherapie sowie der gängigen glaukomchirurgischen Methoden zusammengefasst. Die verschiedenen Wirkansätze der neuen minimal-invasiven Verfahren werden erläutert, die derzeit in Deutschland am häufigsten genutzten Verfahren vorgestellt sowie Empfehlungen für Vor- und Nachsorge gegeben.

**Ergebnisse und Diskussion:**

Seit einigen Jahren stehen außer verschiedenen medikamentösen Tropftherapien und den klassischen operativen Verfahren, auch neue minimal-invasive Therapiealternativen zur Verfügung. Letztere eröffnen die Möglichkeit einer früheren chirurgischen Intervention, besonders für Patienten, die bereits initial oder im Laufe der Therapie für einen medikamentösen Ansatz ungeeignet erscheinen.

## Abstract (en)

**Background:**

The main goals of glaucoma treatment are to preserve the visual function and maintain as high a quality of life as possible at a cost acceptable to society. Therefore, it is crucial to carefully observe each individual patient in order to determine an individual and personalized treatment approach.

**Material and methods:**

This article summarizes the advantages and disadvantages of medicinal glaucoma treatment as well as traditional methods of glaucoma surgery, based on the current state of knowledge. The article explains the various mechanisms of action of new minimally invasive procedures, introduces the methods mostly commonly used in Germany and gives recommendations for preoperative care and postoperative follow-up.

**Results/conclusion:**

In addition to the plethora of medicinal glaucoma treatments and classical surgical procedures, new minimally invasive treatment alternatives have become available in the past few years. The latter are an option for an earlier surgical intervention, especially in naïve or previously treated patients who appear to be unsuitable for medicinal treatment.

Die Prävalenz des Glaukoms beträgt in Deutschland 1,3 % im Alter von 35 bis 74 Jahren. Im Jahr 2017 litten schätzungsweise 923.000 Menschen am Glaukom. Im Vergleich zum Jahr 2002 wurde ein Zuwachs von 24 % beobachtet [1]. Für das Jahr 2030 sollen diese Zahlen auf 1,18 Mio. bzw. 1,63 Mio. Fälle steigen [2]. Das Glaukom ist die zweithäufigste Erblindungsursache weltweit. Das Ziel der Behandlung bei Glaukompatienten ist es, die Sehfähigkeit zu bewahren und damit eine möglichst hohe Lebensqualität zu erhalten. Hierbei ist es wichtig, jeden einzelnen Patienten sorgfältig zu evaluieren, um einen individuellen und personalisierten Therapieansatz zu finden.

Wir wissen aus Langzeitdaten, dass Patienten mit einem intraokularen Druck (IOD) unter 18 mm Hg über einen Zeitraum von 6 Jahren keine weitere Zunahme an Gesichtsfelddefekten zeigen [3]. Ein individueller Zieldruck basiert auf dem festgestellten Glaukomschaden, dem Ausgangsdruck, der Progressionsrate, der Durchblutungssituation und genetischer Disposition sowie dem Alter und der voraussichtlichen Lebenserwartung des Patienten. Danach wird eine entsprechende Behandlung angesetzt.

## Vor- und Nachteile medikamentöser Glaukomtherapie

Der Schwerpunkt im Schema der Empfehlungen der European Glaucoma Society (EGS) aus dem Jahr 2014 liegt auf einer konsequenten medikamentösen Einzel- oder Kombinationstherapie, die nicht notwendigerweise für alle Glaukomarten oder alle Patienten anwendbar sein dürfte. Eine Unterscheidung zwischen konservierten und unkonservierten Darreichungsformen findet noch nicht statt. Zudem sind neuere, nichtmedikamentöse Optionen wie minimal-invasive Glaukomchirurgie (MIGS) nicht erwähnt, da zum Zeitpunkt des Erscheinens die Datenlage hierzu noch lückenhaft war [4].

Eine Metaanalyse von 114 randomisierten kontrollierten Studien aus dem Jahr 2016 kommt zu dem Schluss, dass die in Augentropfen eingesetzten Prostaglandinanaloga über die stärksten drucksenkenden Eigenschaften verfügen [5].

Da es sich bei der medikamentösen Glaukomtherapie um eine Langzeitbehandlung handelt, ist die Mitarbeit der Patienten unabdingbar. Die Schwierigkeit, diese zu erreichen, liegt u. a. an der Symptomlosigkeit des Glaukoms im frühen Krankheitsstadium, in dem beginnende Gesichtsfelddefekte noch nicht wahrgenommen bzw. durch das Partnerauge kompensiert werden. Eine 2006 veröffentlichte Studie untersuchte das Verhalten von Patienten hinsichtlich ihrer Therapietreue (Adhärenz). Es zeigte sich, dass 50 % der neu mit primärem Offenwinkelglaukom (POWG) diagnostizierten Patienten nicht zu Folgebesuchen erschienen. Innerhalb von 6 Monaten wurde die Hälfte aller Rezepte mit verordneten IOD-senkenden Tropfen nicht mehr in der Apotheke eingelöst. Im Vergleich zu anderen Wirkstoffgruppen schnitten die Prostaglandinanaloga bei der Adhärenz noch am besten ab. Nach 24 Monaten wendeten allerdings auch dort nur noch ca. 30 % der Patienten ihre Therapie an [6].

Eine aktuelle Studie untersuchte die Adhärenz beim Einlösen von Rezepten anhand einer repräsentativen Stichprobe von gesetzlich versicherten Glaukompatienten. Im Zeitraum eines Jahres lag der mittlere Wert bei 66,5 %. Von einer Nichtadhärenz waren besonders folgende Gruppen betroffen: Patienten zwischen 50 und 59 Jahren sowie über 80-jährige, Patienten mit einer längeren Erkrankungsdauer, Patienten mit Betreuungsbedarf sowie Patienten mit mindestens 3 zusätzlichen schweren Erkrankungen [7].

Im Alter kommt es oft zu einer Verschlechterung der feinmotorischen und kognitiven Fähigkeiten, welche die Applikation von Augentropfen erschwert, was sich wiederum auf die Adhärenz auswirken kann [8]. Derzeit erhältliche Tropfhilfen können die Anwendung erleichtern, sie erfahren aber wenig Akzeptanz beim Patienten, und das vielfältige Angebot ist unübersichtlich.

Ein weiterer Faktor ist die Anzahl der zu verabreichenden Medikamente sowie die Häufigkeit der täglichen Anwendung: Je weniger Tropffläschchen ein Patient verwendet, umso besser seine Adhärenz. Im Rahmen einer Studie mit Kombinationstherapien berichteten u. a. 35 % der Patienten von Schwierigkeiten, die Augentropfen zu applizieren, 14 % kamen mit dem Tropffläschchen nicht zurecht [9]. Während schwere systemische Nebenwirkungen bei Anwendung von topischen Drucksenkern der Wirkstoffkategorie Prostaglandinanaloga eher gering sind, mindern v. a. die lokalen Begleiterscheinungen die Therapieadhärenz; ein Beispiel hierfür wären die auftretenden Hyperämien. In den meisten Mehrdosenbehältnissen wird Benzalkoniumchlorid (BAK) als Konservierungsmittel eingesetzt, das bei längerer Anwendung die Stabilität des Tränenfilms herabsetzen und Symptome eines trockenen Auges verursachen oder verschlimmern kann. Auch kann es zu Veränderungen in den tieferen kornealen Schichten kommen, was zu erheblichen Schäden an der Hornhaut führt [10]. Teilweise werden durch BAK auch Allergien verursacht. Konservierungsmittelfreie Augentropfen sind prinzipiell schonender und eignen sich daher eher für Glaukompatienten. Sie werden oft in Einzeldosen angeboten, die jedoch aufgrund der Größe der Ophtiolen schwer zu applizieren sind. Eine Verwendung als Ersttherapie ist bei empfindlichen Patienten auf jeden Fall zu bevorzugen und sollte auch bei weniger empfindlichen Patienten erwogen werden, um einer zukünftigen Schädigung der Augenoberfläche vorzubeugen. Aber auch über die nichtmedikamentösen Möglichkeiten muss der Patient aufgeklärt werden [11].

## Glaukomchirurgie – effektiv, komplex, aufwendig

Gemäß aktueller Empfehlungen aus Schweden und England kann der Einsatz von operativen Verfahren bereits als initiale Therapie oder sehr früh nach Beginn einer medikamentösen Therapie erwogen werden, wenn der Patient einen sehr hohen Ausgangsdruck hat, sich früh eine Progression einstellt [12], bei andauernder Unverträglichkeit der medikamentösen Alternativen [13] oder wenn offensichtlich keine Therapietreue vorliegt.

Bei den chirurgischen Interventionen werden filtrierende Verfahren und nicht filtrierende Verfahren unterschieden.

Die Trabekulektomie stellt weiterhin den Referenzstandard der filtrierenden Verfahren dar. Sie ist das bewährteste Verfahren für die Behandlung des Offenwinkelglaukoms. Die Langzeitergebnisse dieser Methode zeigen zu 80 % eine ausreichende Drucksenkung nach 20 Jahren [1, 2]. Der Erfolg der Operation ist auch von der Erfahrung des Operateurs abhängig [14]. Die meisten Operationen erfolgen unter dem Einsatz von Antimetaboliten (Mitomycin C). Langzeitdaten zeigen, dass viele Patienten nach der Operation zumeist ohne weitere medikamentöse Therapie bleiben. Prinzipiell können sehr niedrige Druckwerte erreicht werden [15]. Im Rahmen einer aktuellen Studie unterzogen sich Patienten mit unkontrolliertem Glaukom erstmalig einem chirurgischen Eingriff. In der Gruppe, die eine Trabekulektomie mit Mitomycin C erhielten (*n* = 117), lag die kumulative Misserfolgsrate nach 3 Jahren bei 28 %, der mittlere IOD bei 12,1 ± 4,8 mm Hg und die Zahl der zusätzlichen Medikation bei 1,2 ± 1,5. Bei 8 % der Patienten traten schwerwiegende Komplikationen auf [16].

Eine im April 2020 publizierte Studie verglich den Erfolg der Trabekulektomie mit der der XEN®-Gelstent-Implantation (Allergan, Dublin, Irland). Das primäre Zielkriterium (Tensio <18 mm Hg ohne Therapie, keine Revision im ersten Jahr) wurde in beiden Gruppen nahezu gleich häufig erreicht. Die absolute Drucksenkung war jedoch in der Trabekulektomiegruppe höher [17].

Einfluss auf den Erfolg der Operation nimmt in großem Maße die Bindehautsituation präoperativ. Eine langjährige Tropfenapplikation führt zu einer nachgewiesenen Entzündung der Bindehaut u. a. mit einer Zunahme an Lymphozyten, Mastzellen und Fibroblasten. Darum ist es empfehlenswert, die lokale antiglaukomatöse Tropftherapie vor der Operation abzusetzen und eine Regeneration der Augenoberfläche zu ermöglichen [4]. Die additive Gabe von steroidhaltigen Augentropfen oder zumindest nichtsteroidaler Antiphlogistika präoperativ erhöht die Langzeitprognose der Sickerkissenfunktion. Während der Operation kommt es bei einem gut vorbereiteten, reizfreien Auge und erfahrenem Operateur nur selten zu einer ernsthaften Komplikation. Bei dünner Bindehaut ist besondere Vorsicht bei der Präparation derselben geboten. Die Iridektomie sollte möglichst klein und peripher angelegt werden, um unnötige Blendungsempfindlichkeiten zu vermeiden.

Heutzutage sehr selten geworden ist die hämorrhagische Aderhautschwellung, die am häufigsten in der postoperativen Periode (1. bis 3. postoperativer Tag) auftritt und v. a. bei Patienten in höherem Alter, bei hoher Myopie, kongenitalem Glaukom und Zustand nach Vitrektomie auftreten kann.

Eine vorübergehende postoperative Visusminderung aufgrund der Refraktionsänderung und Astigmatismusinduktion ist üblich und sollte im Aufklärungsgespräch vermerkt werden. Eine intensive postoperative Tropfentherapie (ggf. Atropin, Steroide) sowie eine mögliche Epitheliopathie bei 5‑Fluorouracil-Nachbehandlung und notwendige Suturolysen können das Sehen eine Zeit lang beeinträchtigen. Es sollte darauf hingewiesen werden, dass sich der zu erreichende Zieldruckbereich erst im Laufe der Zeit einstellen wird und dass postoperativ unmittelbar Maßnahmen ergriffen werden (Suturolysen, Sickerkissen-Steppings oder 5‑FU-Injektionen), um die Wundheilung zu modulieren und das Sickerkissen langfristig funktionsfähig zu erhalten [18, 19].

Sicher ist, dass die Trabekulektomie weiterhin die effektivste Operation zur Behandlung des Glaukoms ist und auch nicht durch die Weiterentwicklungen und neuen Verfahren abgelöst wurde, wie vielleicht anfangs vermutet.

## Minimal-invasive Glaukomchirurgie – „The missing link“

Wie auch bei den konventionellen filtrierenden Verfahren sollte nach sorgfältiger Beurteilung des Patienten in von der EGS empfohlener Weise ein individueller Zieldruck festgelegt werden. Seine Bestimmung stützt sich auf das Krankheitsstadium, die Progression der Erkrankung, das Lebensalter sowie zusätzliche Risikofaktoren. Die EGS-Empfehlungen beinhalten eine Richtschnur für die Drucksenkung in verschiedenen Glaukomstadien. So sollte bei einem frühen Glaukom eine IOD-Senkung von mindestens 20 % (IOD unter 21 mm Hg), bei einem mittleren Glaukom von mindestens 30 % (IOD unter 18 mm Hg) und bei einem fortgeschrittenen Glaukom eine noch stärkere IOD-Senkung erreicht werden [4].

Seit einigen Jahren stehen verschiedene, teils minimal-invasive Ab-interno-Verfahren und Stents zur Verfügung, die mit einem geringeren Risiko der Überfiltration einhergehen. Der Einsatz dieser neuen Verfahren ist in diesem Konzept noch nicht berücksichtigt. Die minimalinvasiven Techniken (MIGS) umfassen Verfahren oder implantierbare Medizinprodukte. Die Eingriffe sollten die folgenden 5 Eigenschaften vereinen:

- Mikroinzision durch die Kornea. Hierbei wird die Bindehaut geschont und daraus folgend eine Narbenbildung derselben vermieden.
- Eingriff ist minimal traumatisch für das Gewebe. Die eingesetzten Produkte verfügen über eine sehr gute Biokompatibilität und unterstützen oft die biologischen Abflusswege des Kammerwassers.
- Effiziente Drucksenkung ist aufgrund der Intervention zu erwarten.
- Sehr gutes Sicherheitsprofil, insbesondere im Hinblick auf die möglichen Komplikationen, wie sie bei den klassischen chirurgischen Verfahren auftreten können.
- Schnelle postoperative Rehabilitation führt zu einer minimalen Beeinträchtigung der Lebensqualität des Patienten. Dieser Aspekt kann eine bessere Akzeptanz durch den Patienten bewirken, und auch der konservativ tätige Augenarzt kann den Patienten ohne erhöhten Aufwand betreuen.

Die drucksenkenden Eigenschaften der meisten MIGS-Verfahren sind in der Regel weniger ausgeprägt als bei den bereits erwähnten klassischen Ab-externo-Operationen wie der Trabekulektomie. MIGS stellen einen Kompromiss dar, den man aufgrund des oben ausgeführten sehr günstigen Risikoprofils eingeht. Daher wird die MIGS in einem frühen Krankheitsstadium präferiert. Dies bedeutet aber auch, dass v. a. Patienten mit fortgeschrittenem Glaukom oder Patienten, für die ein sehr niedriger postoperativer IOD angezeigt ist, nicht zu den eigentlichen Zielkandidaten für MIGS gehören [20].

**Figure 1:**
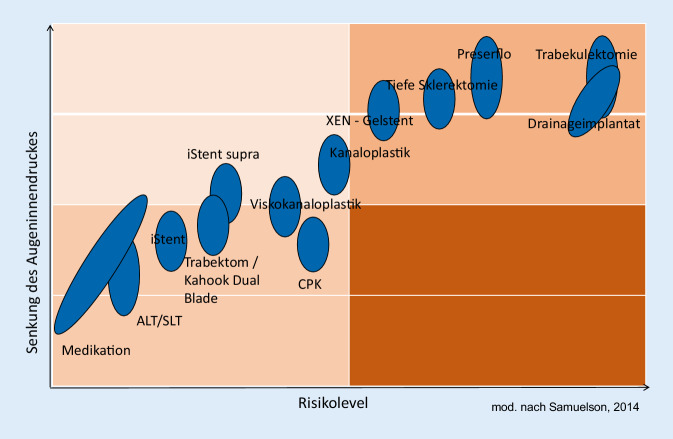

Für die einzelnen Interventionen kann eine Relation zwischen dem Risiko der Methode und der Effektivität der IOD-Senkung hergestellt werden (Abb. 1; [21]).

Die MIGS-Verfahren können in 3 anatomische Kategorien unterteilt werden: Die erste Kategorie adressiert den Schlemm-Kanal für eine Verbesserung des trabekulären Abflusses. Sie nutzt den bestehenden physiologischen Abflussmechanismus und senkt den IOD moderat, da die maximale Senkung durch den episkleralen Venendruck begrenzt wird. Das Risiko einer Hypotonie nach dem Eingriff ist gering, es kann zu peripheren anterioren Synechien kommen. Der Einsatz ist für Patienten mit leichtem bis moderatem Glaukom empfohlen sowie für Patienten mit niedrigen Ausgangsdruckwerten, Patienten mit mehreren medikamentösen Drucksenkern, Patienten, die Unverträglichkeiten zeigen und/oder deren Therapietreue beeinträchtigt ist. Die zweite Kategorie der suprachoroidalen MIGS verbessert den uveoskleralen Abflussmechanismus und hat wahrscheinlich ein höheres drucksenkendes Potenzial. Mögliche Begleiterscheinungen umfassen ein höheres Risiko der vorübergehenden Hypotonie und eines Hyphämas. Der Einsatz wird ebenfalls bei Patienten mit leichtem oder moderatem Glaukom empfohlen und v. a. bei Patienten mit höherem Ausgangsdruck. Die dritte Kategorie der subkonjunktivalen MIGS eröffnet eine alternative Abflussmöglichkeit des Kammerwassers in den Subkonjunktivalraum. Im Verlauf des Eingriffs wird ein Sickerkissen ausgebildet. Um nach der Operation die Bildung von Fibrosen zu verhindern, erfolgt meist der Einsatz von Mitomycin C (MMC). Die Komplikationen ähneln denen der offenen filtrierenden Verfahren, wie z. B. der Trabekulektomie. Die Anwendung wird empfohlen bei Patienten mit weiter fortgeschrittenem Glaukom sowie Patienten mit höheren Ausgangsdruckwerten, bei denen ein niedrigerer Zieldruck erreicht werden soll. Auch Patienten, bei denen mit den ersten beiden Kategorien der MIGS keine erfolgreiche IOD-Kontrolle erreichbar war, könnten für den subkonjunktivalen Ansatz infrage kommen [22].

Eine Auswahl der aktuell wichtigsten MIGS ist in Tab. 1 dargestellt.

**Table 1:** 

Verfahren/Produkt	iStent®	iStent® inject/iStent W	Hydrus® microstent	iStent® supra	XEN®	PreserFlo®/SIBS (Zuvor InnFocus microshunt®)
*Ableitungsweg des Kammerwassers*	Trabekulär	Trabekulär	Trabekulär	Suprachoroidal	Subkonjunktival	Subkonjunktival
*Hersteller*	Glaukos Inc., USA	Glaukos Inc., USA	Ivantis Inc., USA	Glaukos Inc., USA	Allergan plc., Irland	Santen, Japan
*Mechanismus*	Produkt wird durch das Trabekelwerk in den Schlemm-Kanal eingeführt	Produkt wird durch das Trabekelwerk in den Schlemm-Kanal eingeführt	Intrakanalikuläres Gerüst wird in den Schlemm-Kanal eingeführt	Kontrollierte Zyklodialyse mit einem Stent zum Abfluss in suprachoroidalen Raum	Abfluss aus der Vorderkammer in den subkonjunktivalen Raum	Abfluss aus der Vorderkammer in den subkonjunktivalen/subtenonalen Raum
*Material*	Heparin-beschichtetes Produkt aus nicht ferromagnetischem Titan (0,3 mm hoch und 1 mm lang)	Heparin‐beschichtetes Produkt aus nicht ferromagnetischem Titan (340 µm lang, 230/400 µm Durchmesser)	Nickel-Titan-Legierung (Nitinol, 8 mm lang)	Polyethersulfon und Titan (4 mm lang, Lumen: 0,16–0,17 mm)	Kollagenbasierte Schweinegelatine quervernetzt mit Glutaraldehyd (6 mm lang, Lumen: 45 µm)	Thermoplastisches Elastomer „SIBS“ („polystyrene-block-isobutylene-block-styrene“) (8,5 mm lang, Lumen 70 µm)
*Produktinformationen*	Medizinprodukt mit CE-Kennzeichnung Indikation: POWG, PEX oder Pigmentglaukom	Medizinprodukt mit CE-Kennzeichnung Indikation: POWG, PEX oder Pigmentglaukom	Medizinprodukt mit CE-Kennzeichnung IOD-Reduktion bei leichtem bis mittelschweren POWG	Medizinprodukt mit CE-Kennzeichnung Indikation: POWG, PEX oder Pigmentglaukom	Medizinprodukt mit CE-Kennzeichnung Indikation: refraktäres Glaukom, nicht medikamentös therapierbar	Medizinprodukt mit CE-Kennzeichnung Indikation: POWG (leicht bis moderat)

Minimal-invasive Verfahren stellen derzeit eine Nischenanwendung im anerkannten Behandlungsalgorithmus beim Glaukom dar. Die noch fehlende Langzeiterfahrung muss jedoch kein Ausschlusskriterium für den Einsatz der MIGS-Verfahren darstellen. Neben den Daten aus wenigen randomisierten kontrollierten klinischen Prüfungen existieren viele prospektive und retrospektive Fallserien, v. a. für die trabekulären und subkonjunktivalen Varianten. Um den am häufigsten verwendeten Vertreter der suprachoroidalen MIGS (iStent® supra aus Polyethersulfon und Titan und das mikroporöse Silikonimplantat MINIject [R]) zu beurteilen, stehen bisher nicht genügend bzw. noch keine Daten zur Verfügung.

Die Wahl des MIGS-Verfahrens sollte auch die persönliche Situation des Patienten berücksichtigen. Die Unabhängigkeit von einer medikamentösen Dauertherapie oder die Reduktion der Medikation ist dabei auch ein Ansatz zur Verbesserung von bekannten Adhärenzproblemen und damit Steigerung der Lebensqualität.

Der Erfolg bei der Verwendung der MIGS hängt nicht zuletzt von der Qualität der Chirurgie oder der postoperativen Begleitung des Patienten ab. Für Ersteres ist eine sorgfältige Ausbildung des Operateurs erforderlich. Die Glaukomchirurgie ist keine Trivialchirurgie. Dabei haben sich eine Einführung unter Begleitung eines erfahrenen Anwenders sowie der kontinuierliche Erfahrungsaustausch unter Anwendern bewährt. Je komplexer die operative Technik, desto länger ist die Lernkurve. Dies gilt v. a. für die Anwendung der subkonjunktivalen Techniken wie des XEN® Gelstents oder des Preserflo® microshunts, bei denen die Positionierung und das postoperative Management anspruchsvoller sind.

Während die unmittelbare postoperative Begleitung noch beim Operateur liegt, erfolgt die weitere Verlaufskontrolle dann wieder beim Zuweiser. Hierfür ist auch eine enge Kommunikation zwischen beiden Akteuren wichtig. Auch der Zuweiser sollte genaue Kenntnisse über das verwendete Verfahren sowie die daraus möglicherweise resultierenden Begleiterscheinungen und deren Interpretation und Behandlung besitzen.

**Figure 2:**
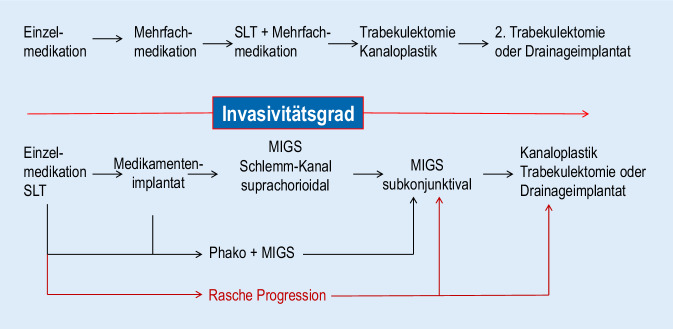

Die weniger invasiven Verfahren sollten als weitere sinnvolle Ergänzung der anderen operativen Optionen verstanden werden. Sie können zum Einsatz kommen, wenn ein frühes Glaukom vorliegt, bei Patienten mit Tropfentherapie eine Verschlechterung des Gesichtsfelds beobachtet wird oder der Patient konventionelle operative Verfahren ablehnt. Die klassischen Verfahren haben weiter ihren Platz im Behandlungsablauf. Operative Verfahren insgesamt kommen für Patienten mit geringen bis moderaten Gesichtsfelddefekten in Betracht, wenn die medikamentöse Therapie keine ausreichende Drucksenkung bewirkt bzw. der IOD über dem Zieldruck liegt, die Patienten nicht oder vermutet nicht therapietreu sind und somit das Progressionsrisiko erhöht ist. Ebenso sollte der Einsatz bei früher oder schneller Progression erwogen werden oder wenn für den Patienten ohnehin eine Kataraktoperation ansteht [12]. Die den subkonjunktivalen oder suprachoroidalen Raum filtrierenden Verfahren können möglicherweise auch als Ersatz für eine Trabekulektomie fungieren. Ein zukünftiger Behandlungsalgorithmus ist in Abb. 2 dargestellt: Therapieoptionen nach Intensitätsgrad der Intervention [21].

## Trabekuläre Verfahren

Der wichtigste Vertreter der trabekulären MIGS ist der iStent® inject bzw. sein Vorgängermodell iStent®. Der Stent besteht aus nicht ferromagnetischem Titan, das mit Heparin beschichtet ist. In einer Vielzahl von Studien wird nur über die Wirkung des Stents in Kombination mit einer Kataraktoperation berichtet. Die Drucksenkung liegt beim Heparin-beschichteten Titan-Stent (iStent® inject, dem neueren iStent inject® W sowie sein Vorgängermodell iStent®) bei 15–25 % [23]. Eine aktuelle Publikation mit 2‑Jahres-Daten kam zu dem Schluss, dass die IOD-Senkung ohne zusätzliche Medikation mit iStent® inject in Kombination mit einer Kataraktoperation klinisch und statistisch signifikant größer ist als bei einer alleinigen Kataraktoperation: Nach 24 Monaten hatten immer noch 75,8 % der mit iStent® inject behandelten Augen eine mehr als 20 %ige Reduktion vom Ausgangsdruck, die mittlere IOD-Senkung lag bei 7,0 ± 4,0 mm Hg und 63,2 % der Augen hatten einen IOD von maximal 18 mm Hg [24]. Im Rahmen der Synergy-Studie an 99 Patienten wurde die Wirkung des iStent® inject als Standalone-Therapie an Patienten dokumentiert, die zuvor mindestens 2 IOD-Senker erhalten hatten. Nach 12 Monaten hatten 66 % einen IOD von maximal 18 mm Hg ohne zusätzliche Medikation. Der mittlere Anfangsdruck konnte von 26,3 mm Hg (SD 3,5) auf 15,7 mm Hg (SD 3,7) bzw. um 39,7 % gesenkt werden [25].

Der Hydrus® microstent ist 8 mm lang und besteht aus einem Nitinol-Gerüst, das in den Schlemm-Kanal eingeführt wird. Bei der Mehrzahl der durchgeführten Studien wird der Stent in Kombination mit einer Kataraktoperation (Phako) eingesetzt [26]. Eine randomisierte klinische Studie aus dem Jahr 2015 verglich die IOD-Senkung des Hydrus® zusätzlich zur Kataraktoperation mit der alleinigen Kataraktoperation: Nach 24 Monaten hatten signifikant mehr Patienten mit Hydrus® und Kataraktoperation eine 20 %ige Senkung vom Ausgangsdruck (80 % vs. 46 %, *p* = 0,0008), auch der mittlere IOD war signifikant niedriger (16,9 ± 3,3 mm Hg vs. 19,2 ± 4,7 mm Hg, *p* = 0,0093) und der Anteil an Patienten, die keine zusätzlichen IOD-Senker benötigten (73 % vs. 38 %, *p* = 0,0008) [27].

Dennoch muss beachtet werden, dass die tatsächliche Drucksenkung durch den Hydrus-Stent zusätzlich zur Kataraktoperation den Augeninnendruck nur etwa 2 mm Hg mehr senkt als ohne Stent.

In einer weiteren randomisierten kontrollierten Studie (*n* = 152) wurde die Wirkung des Hydrus® mit der Applikation von 2 iStents® über einen Zeitraum von 12 Monaten verglichen. Die Zielkriterien für eine erfolgreiche Behandlung umfassten einen IOD bis 18 mm Hg, keine zusätzliche IOD-senkende Medikation, keine sekundäre Glaukomchirurgie, Trabekuloplastik oder Kataraktoperation. In der Hydrus®-Gruppe erreichten 36 % dieses Ziel, in der iStent®-Gruppe nur 10,5 %. Nach 12 Monaten lag der mittlere IOD bei jeweils 17,3 ± 3,7 mm Hg bzw. 18,1 ± 3,7 mm Hg und die Zahl der IOD-senkenden Medikamente bei 1,0 bzw. 1,7. Die Rate der Reoperationen war in beiden Gruppen niedrig (0 bzw. 2,6 %) [28].

## Subkonjunktivale Verfahren

Das subkonjunktivale Gelimplantat (XEN®) ist 6 mm lang und besteht aus kollagenbasierter Schweinegelatine. Bei einem Druckgradienten von 5 mm Hg fließt durch das Gelimplantat 1,2 μl/min ab, sodass ein Abflusswiderstand von ca. 6–8 mm Hg erreicht wird. Das Implantat ist nicht abbaubar und wird über einen beladenen Injektor ab interno appliziert.

In Deutschland ist der Einsatz des Gelimplantats beim POWG indiziert, wenn eine medikamentöse Therapie nicht ausreicht. Bevorzugt könnten Patienten mit POWG, Pseudoexfoliationsglaukom (PEX), Pigmentglaukom oder Normaldruckglaukom behandelt werden. Die Höhe des Ausgangsinnendrucks hat keine Auswirkung auf den Erfolg der Operation, und der Stent ist auch bei initialen Drücken von 40–50 mm Hg gut geeignet [29]. Auch Patienten, die bereits eine Trabekulektomie oder ein großes Drainageimplantat hatten, können geeignet sein, sofern der nasal obere oder untere Quadrant frei ist. Der Eingriff kann bei Bedarf mit einer Kataraktoperation kombiniert werden [30].

Für die Anästhesie gibt es keine konkreten Vorgaben. Sie kann sowohl als Tropfanästhesie als auch subkonjunktival oder parabulbär erfolgen. Das Implantat sollte so positioniert werden, dass der Abfluss des Kammerwassers nach nasal oben erfolgt. Ein Stichkanal von ca. 3 mm wird empfohlen, funktioniert aber nicht in jedem Fall. Ein zu kurzer Stichkanal kann zu einem erhöhten Hypotonierisiko führen.

Die Applikation von MMC erfolgt in der Regel mit einer Injektion, die mindestens 5–6 mm von Limbus in Richtung des zu erwartenden Filterkissens gesetzt wird. Am häufigsten werden 5–20 µg MMC injiziert. Die Vor- und Nachteile beim Einsatz von Antimetaboliten werden im folgenden Kapitel besprochen.

Sobald das Implantat über den Injektor abgesetzt ist, muss überprüft werden, ob es sich frei in der Schicht bewegen kann (radiäre Sitzform). Ist die Spitze gebogen oder nicht frei beweglich, dann soll versucht werden, die Lage transkonjunktival zu korrigieren. Bei ausbleibendem Erfolg kann auch ein „primäres Needling“ mit einer 30-G-Kanüle durchgeführt werden. Um eine genaue Positionierung des Implantats im Kammerwinkel zu erreichen, wird eine intraoperative Gonioskopie empfohlen. Nach Überprüfen der korrekten Eintrittsstelle oberhalb des Trabekelmaschenwerkes erfolgt das Einsetzen des Implantats. Für das Gelimplantat liegen zahlreiche prospektive und retrospektive Studien vor [31–33].

Andauernde Hypotoniekomplikationen wie eine hypotone Makulopathie oder eine ausgeprägte Aderhautamotio sind nicht beschrieben worden. Allerdings gibt es einen Fallbericht über eine hämorrhagische Aderhautblutung nach XEN-Gelstent-Implantation in der Literatur [34]. Außerdem ist die Migration des Stents unter der Bindehaut und auch in der Vorderkammer beschrieben [35, 36].

Im Rahmen der aktuellsten prospektiven internationalen 2‑Jahres-Studie wurde das Gelimplantat bei 200 Augen auch im Rahmen einer Kataraktoperation eingesetzt. Die Drucksenkung lag bei 30–45 %. Am Studienende benötigten 45 % der Patienten keine drucksenkende Medikation [32]. Etwa ein Drittel der Patienten braucht im Verlauf ein Needling [30, 32].

Eine weitere subkonjunktivale Option stellt ein Shunt aus dem thermoplastischen Elastomer „styrene-block-isobutylene-block-styrene“ („SIBS“, Preserflo®) dar. Die Operation ist eigentlich eine Shunt-geführte Trabekulektomie. Nach Eröffnung der Bindehaut wird 3 mm hinter dem Limbus eine 1 mm breite Skleraltasche mit einem abgewinkelten Messer präpariert. Eine 25-G-Nadel wird dann durch die Skleraltasche in die Vorderkammer geführt. Der Shunt wird vorgeschoben, und die Flügel kommen in der Skleratasche zu liegen. Danach erfolgt eine Tenon- und Bindehautnaht. Die Operation ist technisch weniger anspruchsvoll als eine Trabekulektomie oder eine XEN®-Gelstent-Implantation. Die OP-Dauer ist in etwa vergleichbar mit der Trabekulektomie, da sowohl für die Mitomycin-Einlage als auch für die Vorbereitung der Skleratasche und des Kanals Zeit benötigt wird. Die Vorsorge entspricht der der Trabekulektomie. Postoperativ können 5‑FU-Injektionen zur Wundmodulation gegeben werden [37].

Daten aus einer kleineren Beobachtungsstudie mit Preserflo® belegen eine Drucksenkung von 53 % [38]. Eine multizentrische Studie an 91 Augen hat gezeigt, dass mit Preserflo® der mittlere Ausgangsdruck nach 1 Jahr um 45 % gesenkt werden konnte (von 24 ± 5,9 auf 13,3 ± 4,0 mm Hg), wobei 90 % aller Patienten einen IOD unter 18 mm Hg hatten und 83 % keine zusätzlichen Drucksenker benötigten [39]. Die Ergebnisse dieser 24-monatigen, prospektiven, randomisierten, kontrollierten multizentrischen Studie, die die Wirksamkeit und Sicherheit von Preserflo® mit der Trabekulektomie vergleichen, werden in 2020 erwartet [40].

## Expert Principles – Überlegungen vor und nach der MIGS

Konkrete Empfehlungen, welche MIGS-Verfahren für welche Patientenpopulation einzusetzen sind, existieren derzeit noch nicht. Zunächst ist der Patient auch hier hinsichtlich der oben bereits erwähnten Risikofaktoren der EGS-Richtlinien zu überprüfen. Nach einer eingehenden Beratung sollte das Ziel sein, eine sog. „supported medical decision“ zu erreichen d. h. eine Therapieentscheidung, die vom Patienten dann auch mitgetragen wird. Ist die Entscheidung für den Einsatz einer MIGS gefallen, sind je nach Art des Implantats in Vorbereitung auf den Eingriff, während der Intervention sowie bei der Nachsorge bestimmte Schritte zu beachten (Tab. 2; [41]).

**Table 2:** 

Verfahren	Trabekuläre Stents	Subkonjunktivale Verfahren	
*Produkte*	*iStent®, iStent® inject und ’W’, Hydrus® microstent*	*XEN®*	*PreserFlo®/SIBS*
*Präoperative Vorbereitung*	Keine	4 Wochen zuvor: Absetzen konservierter AT, Umstellung auf unkonservierte topische oder systemische Therapien (bessere Sickerkissenbildung) 5 bis 14 Tage zuvor: Gabe von unkonservierten Steroiden (Normalisierung der Bindehaut)	
*Besonderheiten*	Kurze OP Dauer, Kombination mit Kataraktoperation, Wissen über die Anatomie des Kammerwinkels wichtig	Mitomycin C, längere OP Dauer, ohne Bindehauteröffnung	Mitomycin C, längere OP Dauer, mit Bindehauteröffnung
*Nachsorge*	Antibiotika-AT 5 bis 7 Tage Unkonservierte Kortikosteroid-AT über 2 bis 5 Wochen	Postoperative Kontrolle Tag 1 bis 2, im ersten Monat 1–2 pro Woche, nach 6 Wochen und 3 Monaten Unkonservierte Kortikosteroid-AT über 6 bis 8 Wochen (Prednisolon oder Dexamethason) Unkonservierte Antibiotika-AT über 7 Tage IOD, Vorderkammertiefe, Sickerkissenbildung dokumentieren 5‑FU-Gabe möglich Bei Hypotonie und Vorderkammerabflachung Atropin-Gabe möglich	
*Mögliche Komplikationen und Maßnahmen*	Fehlpositionierung des stents, fehlende Drucksenkung, starke intrakamerale Blutung	Siehe Abb. 1 und 2	Keine chronischen Hypotonien zu erwarten Hypertonien: Fibrosierungen des Filterkissens

*AT* Augentropfen

Vor allem die Nachsorge bei den subkonjunktivalen Verfahren (XEN Gelimplantat und PreserFlo ist umfangreicher und muss engmaschiger erfolgen. Hierbei gilt das besondere Augenmerk dem postoperativen Intraokulardruck und der Konfiguration der Filtrationszone. Die Vor- und Nachsorge unterscheidet sich kaum von der Trabekulektomie [42]).

Wenn nach dem Eingriff bei einer korrekten Position eines subkonjunktivalen Implantates der IOD höher als der definierte Zieldruck ist, liegt am ehesten eine Fibrose vor. Je nach Filterkissenkonfiguration unterscheidet sich das weitere Procedere.

Vermutet man im Rahmen der Kontrollen eine beginnende Fibrosierung des Gewebes, kann zunächst eine Gabe von 5‑Fluorouracil (5-FU) oder eines VEGF-Hemmers angezeigt sein. Sollte die Fibrose bereits manifest sein, kann eine invasive Korrektur (Needling, Mobilisation des Implantats) notwendig werden.

## Antimetaboliten (Mitomycin C, 5-Fluorouracil): Noch keine einheitliche Empfehlung

Die Wundheilung, die zu einer Vernarbung führen kann, beginnt bereits direkt postoperativ mit dem Einsprossen von Gefäßen und der Abgabe von Fibrin, Fibronektin und Plasminogen. Im Verlauf migrieren Makrophagen, Monozyten und Fibroblasten. Zur Vernarbungshemmung werden überwiegend die Antimetabolite MMC sowie 5‑FU eingesetzt.

MMC unterbindet die DNA-Replikation und wirkt hemmend in allen Phasen der Zellteilung. Es wirkt ca. 100-mal stärker als 5‑FU. MMC wird als Antimetabolit für die Anwendung im Rahmen der Ab-externo-Glaukomchirurgie in Deutschland seit Jahrzehnten im Off-label-Bereich verwendet. In der Praxis wird MMC einmalig in einer Konzentration von 0,1–0,5 mg/ml bei einer Einwirkzeit zwischen 1 und 5 min verwendet. Eine subkonjunktivale Injektion führt zu ähnlichen Ergebnissen wie die Schwämmchenapplikation [43].

Als alternativer Antimetabolit kann das Zytostatikum 5‑FU verwendet werden. Es hemmt die Zellen nur in der Interphase, und die Wirkung von 5‑FU ist insgesamt schwächer. Die eingesetzte Konzentration liegt bei 5 mg/0,1 ml. Studiendaten zeigten einen Rückgang der Misserfolgsrate von 74 auf 51 % [44]. Die Needlingrate nach Trabekulektomie mit Einsatz von MMC liegt in der Literatur nach 1 bis 2 Jahren bei 16–25 %. Auch beim Gelimplantat scheint der Einsatz von Antimetaboliten die Needlingrate herabzusetzen. Im Rahmen einer Studie wurde das Gelimplantat ohne MMC eingesetzt. Die Needlingrate lag nach 12 Monaten bei 47 % [45]. Im Vergleich hierzu zeigte eine 12-monatige Studie, in der MMC verwendet wurde, dass nur 31 % der Augen ein Needling benötigten [46]. Eine weitere Studie, die das XEN Gelimplantat mit der Trabekulektomie verglich, kam auf Needlingraten von nur 16 % in beiden Gruppen [17]. In der oben bereits erwähnten 2‑Jahres-Studie lag die Needlingrate bei 41 % [32]. Die vorliegenden Daten zeigen, dass der Einsatz von Antimetaboliten die Erfolgswahrscheinlichkeit von operativen Eingriffen wie Trabekulektomie oder subkonjunktivalen Implantaten erhöht. Die Dosierungen sind bislang rein empirisch. MMC wird in den meisten Studien kumulativ in einer Dosis von 10–40 µg eingesetzt.

## Fazit für die Praxis

Vor allem für Patienten mit frühem oder moderat fortgeschrittenem Glaukom bei denen u. U. die Progression unter medikamentöser Kombinationstherapie weiter voranschreitet, kommen MIGS-Verfahren in Betracht. Am wenigsten drucksenkend sind die trabekulären Verfahren. Minimal-invasive Verfahren können auch bei Patienten, die häufig über Nebenwirkungen unter der medikamentösen Therapie klagen, bei Patienten mit trockenem Auge oder solchen, bei denen man eine schlechte Therapietreue annehmen muss, angewendet werden. Nach der derzeitigen Datenlage für MIGS eignen sich die trabekulären und suprachoroidalen Implantate bei frühem Glaukom mit gleichzeitig bestehender Katarakt. Subkonjunktivale Implantate können nach genauer Abwägung gegen den Referenzstandard Trabekulektomie auch bei moderatem und fortgeschrittenem Glaukom angewendet werden. Die Drucksenkung ist oft geringer als bei der Trabekulektomie bei ähnlich hoher Komplikationsrate und gleicher Nachsorge. Der Einsatz von Antimetaboliten ist neben der Trabekulektomie ausschließlich der subkonjunktivalen MIGS vorbehalten. Beim postoperativen Needling kann MMC oder 5‑FU eingesetzt werden. Eine offene Revision ist ebenfalls Erfolg versprechend [44–47].

Für die Verwendung der in Deutschland verwendeten Verfahren liegen inzwischen ausreichend klinische und operative Erfahrungen vor. Randomisierte klinische Studien sind leider nur wenige verfügbar. Die Trabekulektomie bleibt daher aktuell die effektivste Methode für die Behandlung des fortgeschrittenen primären Offenwinkelglaukoms mit niedrigem Zieldruckbereich.
