# Erratum zu: Aktuelle Glaukomchirurgie

**DOI:** 10.1007/s00347-021-01457-7

**Published:** 2021-07-12

**Authors:** Esther M. Hoffmann, Fritz Hengerer, Karsten Klabe, Marc Schargus, Hagen Thieme, Bogomil Voykov

**Affiliations:** 1 Augenklinik und Poliklinik, Universitätsmedizin Mainz, Mainz, Deutschland; 2 Bürgerhospital Frankfurt a. M., Frankfurt a. M., Deutschland; 3 Breyer – Kaymak – Klabe Augenchirurgie, Düsseldorf, Deutschland; 4 Asklepios Augenklinik Nord-Heidberg Hamburg, Hamburg, Deutschland; 5 Universitäts-Augenklinik Düsseldorf, Düsseldorf, Deutschland; 6 Universitätsaugenklinik Magdeburg, Magdeburg, Deutschland; 7 Universitätsaugenklinik Tübingen, Tübingen, Deutschland

**Erratum zu:**

**Ophthalmologe 2021**

10.1007/s00347-020-01146-x

Der Artikel „Aktuelle Glaukomchirurgie“ von Hoffmann, E. M., Hengerer, F., Klabe, K., Schargus, M., Thieme, H. und Voykov, B. wurde ursprünglich Online First ohne „Open Access“ auf der Internetplattform des Verlags publiziert. Nach der Veröffentlichung in Band 118 Heft 3 pp. 239–247 hatten sich die Autoren für eine „Open Access“-Veröffentlichung entschieden. Das Urheberrecht des Artikels wurde deshalb in © Der/die Autor(en) 2020 geändert.

Der Artikel wird nun unter der Creative Commons Namensnennung 4.0 International Lizenz veröffentlicht, welche die Nutzung, Vervielfältigung, Bearbeitung, Verbreitung und Wiedergabe in jeglichem Medium und Format erlaubt, sofern Sie den/die ursprünglichen Autor(en) und die Quelle ordnungsgemäß nennen, einen Link zur Creative Commons Lizenz beifügen und angeben, ob Änderungen vorgenommen wurden.

Die in diesem Artikel enthaltenen Bilder und sonstiges Drittmaterial unterliegen ebenfalls der genannten Creative Commons Lizenz, sofern sich aus der Abbildungslegende nichts anderes ergibt. Sofern das betreffende Material nicht unter der genannten Creative Commons Lizenz steht und die betreffende Handlung nicht nach gesetzlichen Vorschriften erlaubt ist, ist für die oben aufgeführten Weiterverwendungen des Materials die Einwilligung des jeweiligen Rechteinhabers einzuholen.

Weitere Details zur Lizenz entnehmen Sie bitte der Lizenzinformation auf http://creativecommons.org/licenses/by/4.0/deed.de.

